# The antiSMASH database version 3: increased taxonomic coverage and new query features for modular enzymes

**DOI:** 10.1093/nar/gkaa978

**Published:** 2020-11-05

**Authors:** Kai Blin, Simon Shaw, Satria A Kautsar, Marnix H Medema, Tilmann Weber

**Affiliations:** The Novo Nordisk Foundation Center for Biosustainability, Technical University of Denmark, Kgs. Lyngby 2800, Denmark; The Novo Nordisk Foundation Center for Biosustainability, Technical University of Denmark, Kgs. Lyngby 2800, Denmark; Bioinformatics Group, Wageningen University, Wageningen 6708PB, The Netherlands; Bioinformatics Group, Wageningen University, Wageningen 6708PB, The Netherlands; The Novo Nordisk Foundation Center for Biosustainability, Technical University of Denmark, Kgs. Lyngby 2800, Denmark

## Abstract

Microorganisms produce natural products that are frequently used in the development of antibacterial, antiviral, and anticancer drugs, pesticides, herbicides, or fungicides. In recent years, genome mining has evolved into a prominent method to access this potential. antiSMASH is one of the most popular tools for this task. Here, we present version 3 of the antiSMASH database, providing a means to access and query precomputed antiSMASH-5.2-detected biosynthetic gene clusters from representative, publicly available, high-quality microbial genomes via an interactive graphical user interface. In version 3, the database contains 147 517 high quality BGC regions from 388 archaeal, 25 236 bacterial and 177 fungal genomes and is available at https://antismash-db.secondarymetabolites.org/.

## Graphical Abstract

**Graphical Abstract:**
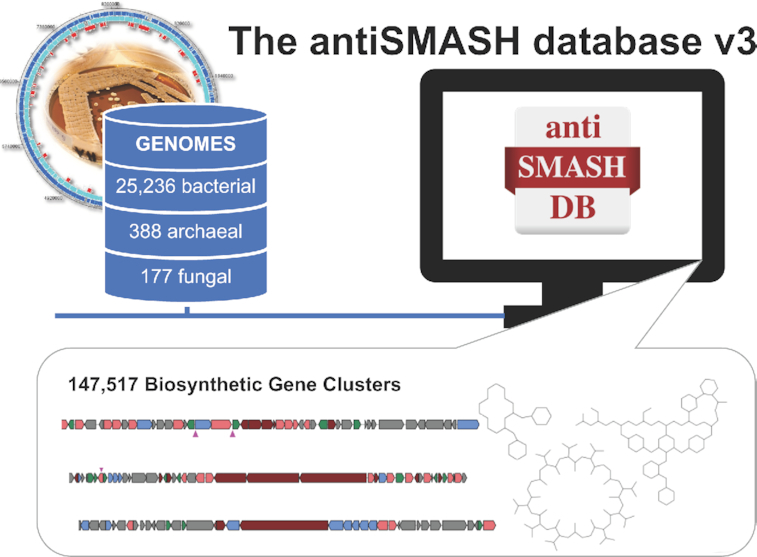
The antiSMASH database was updated to version 3, now containing 147 517 secondary/specialized metabolite biosynthetic gene clusters (BGCs) originating from 25 236 bacterial, 388 archaeal and 177 fungal genomes. The database provides interactive access to the detailed annotation of each BGC that was generated with the most recent version of antiSMASH (v5.2).

## INTRODUCTION

Many drugs, especially drugs with antibiotic or antifungal activity, are based on natural compounds produced by microorganisms (1). The classical approach to identifying new bioactive natural compounds has been to chemically isolate, purify and subsequently test compounds extracted from natural sources. The improved availability of microbial genome data has made it possible to complement this approach with genome mining technologies to identify and characterise natural product biosynthetic pathways from genome and metagenome data (2). Dedicated software to assist researchers in natural product genome mining has been around for over a decade now (please refer to (3–6) for reviews). However, only a few databases, such as ClusterMine360 (7) or the recently updated IMG-ABC (8) exist to make such data available to users.

Since its initial release in 2011, antiSMASH (9–13) has become the most widely used tool for genome mining for secondary/specialized metabolites and is regarded as the gold standard. antiSMASH uses a rule-based approach to detect genome regions containing biosynthetic gene clusters based on conserved biosynthetic enzymes from (currently) 60 different biosynthetic pathways. For BGCs encoding nonribosomal peptide synthetases (NRPS), type I and type II polyketide synthases (PKS), lanthipeptides, thiopeptides, sactipeptides and lassopeptides, antiSMASH performs more in-depth, cluster-type-specific analyses to provide more detailed predictions of biosynthetic steps occurring in the respective biosynthetic gene cluster (BGC), and, by extension, of the compound(s) produced by it. Identified regions can be compared to a database of antiSMASH results predicted on publicly available genomes using the built-in ClusterBlast algorithm. A similar comparison, KnownClusterBlast, is used to compare identified regions against a dataset of manually curated biosynthetic gene clusters with known products from the MIBiG reference database (14,15).

By design, antiSMASH is a genome mining tool that analyses and annotates individual microbial genomes, one at a time. As some research questions can be better answered by an interconnected dataset with cross-genome search capabilities, we developed the antiSMASH database (16,17) to not only make precomputed antiSMASH results for many microbial organisms instantly available, but to also add user-friendly search functionalities on top of that dataset. In addition, the database is used as the basis for antiSMASH’s ClusterBlast functionality, and any ClusterBlast hit links to the database. antiSMASH results in the database thus are cross-referenced to similar other results in the database, as well as to similar clusters from the MIBiG database. Here we present the third version of this database. On top of 25 236 bacterial genomes, this version adds non-bacterial genomes and now also covers 388 archaeal and 177 fungal genomes. Additionally, new query functionalities have been added to search for NRPS and PKS multimodular enzyme systems with architectural features of interest to the user.

## MATERIALS AND METHODS

### Selection of included genomes

While a lot of taxonomically diverse microbial genomes are being published frequently, the NCBI’s genome databases contain a lot of redundancies caused by tens of thousands of sequences, mostly of pathogens such as *Salmonella enterica*, *Escherichia coli* or *Pseudomonas aeruginosa*. To avoid swamping the antiSMASH database with thousands of identical results from strains that differ only by a few single nucleotide polymorphisms, we have previously developed a redundancy filtering/dereplication approach (17) that we have further refined in building the current version of the antiSMASH database.

For archaea and bacteria, we obtained all genomes available on the NCBI RefSeq FTP server with an assembly level of ‘complete’, ‘chromosome’, or ‘scaffold’ in GenBank and FASTA format using the ncbi-genome-download (https://github.com/kblin/ncbi-genome-download/) tool, yielding 94 774 assemblies on 4 September 2020. For fungal genomes, we selected all genomes labeled ‘reference’ or ‘representative’ from RefSeq, and extended the selection by adding all ‘complete’ or ‘chromosome’ level genomes from GenBank. Genomes were again downloaded using the ncbi-genome-download tool and yielded 445 assemblies on August 18th, 2020.

Many natural product BGCs contain repetitive sequences. On low quality draft genomes that consist of many contigs, those clusters are frequently spread across multiple contigs without any linkage information, making it impossible to assemble complete clusters from those low quality data sets. To avoid including assemblies that were too fragmented, we filtered out any assemblies containing >100 contigs.

To filter out redundancies, we again used genomic distance estimations. For fungal sequences, we repeated our previous approach (17), using FastANI (18) to calculate the average nucleotide identity (ANI) between assemblies. ANI values were converted into distances using the formula $d = 1 - \frac{{ani}}{{100}}$, where *d* is the distance and *ani* the similarity percentage value returned by FastANI, and then clustered using scikit-learn's AgglomerativeClustering algorithm (19). The only genomes that clustered at a distance cutoff of ≤0.004 (equivalent to the ≥99.6% ANI we used for the previous version) were the GenBank and RefSeq versions of assemblies that were contained in our dataset twice. In these cases, we used the RefSeq version of that assembly. For bacterial and archaeal sequences, running FastANI on the 71 591 assemblies that survived the ≤100 contigs filter would have been prohibitively expensive in terms of CPU time, so we switched to using the Mash tool (20) to estimate genomic distances instead. Again using a distance cutoff of 0.004 in the clustering steps, the representative genome of each similarity cluster was chosen by picking the assembly with the lowest contig count. If two assemblies had the same contig count, the assembly first occurring in the NCBI download server's assembly_summary.txt file was kept.

### antiSMASH annotations and data import

Using the downloaded genbank files of the representative genomes, antiSMASH 5.2 was run via GNU parallel (21). Different to our previous version (for which we processed all draft genomes in ‘minimal’ mode), all 28 739 dereplicated complete and draft genomes were processed in full antiSMASH runs. In order to build the initial database, a first pass using basic analysis options was run (options: - -cb-knownclusters - -cb-subclusters - -asf). The regions identified during this first pass were extracted, and used to build an updated ClusterBlast database. This updated ClusterBlast database will also be used in future antiSMASH releases. Then, a second pass was run to both include ClusterBlast results based on this new database and also add some more time-intensive analyses (additional options: - -cb-general - -clusterhmmer - -pfam2go). During the antiSMASH annotation phase, all assemblies not containing gene calls were dropped from the dataset (2881 prokaryotic and 57 fungal sequences).

The SQL schema for the database (https://github.com/antismash/db-schema/) was updated to cover antiSMASH 5 annotations. The importer (https://github.com/antismash/db-import/) was rewritten to use antiSMASH 5’s JSON-formatted results file.

## RESULTS AND DISCUSSION

**Figure 1.**
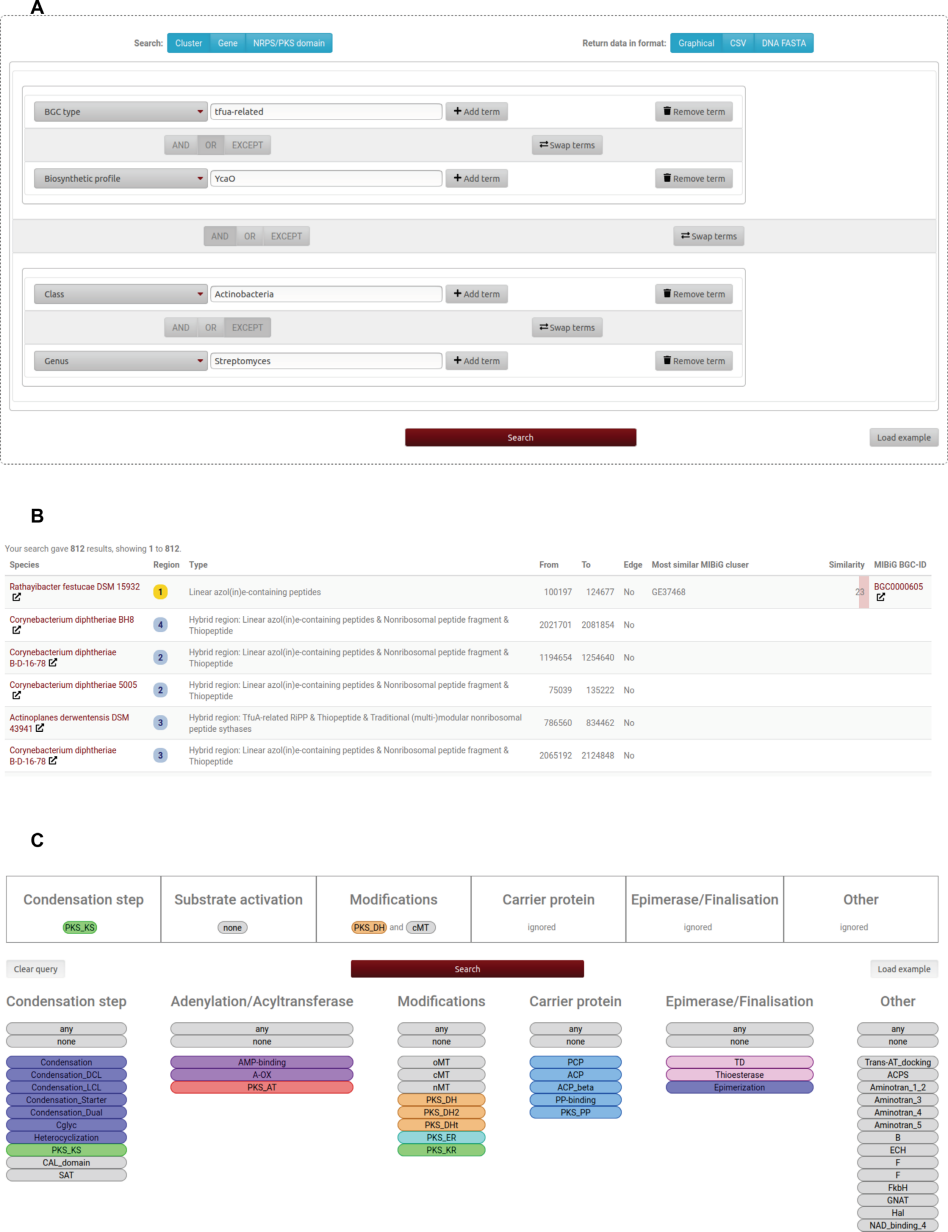
(**A**) Using the query builder to formulate a complex query. In this case, the search is for all TfuA-related RiPPs or any other clusters encoding for the thiolated RiPP-associated YcaO protein in all bacteria of the class Actinobacteria, but not of the genus *Streptomyces*. (**B**) A selection of query results of the query from part A. Hits are found in various *Corynebacterium* sp., but also a number of uncommon actinomycetes. (**C**) Using the module query to search for a *trans*-acyltransferase PKS module that contains both a dehydratase domain and a carbon methyltransferase domain. While the query builder could also be used to search for clusters that contain those two domains, it is not possible to restrict hits to only clusters that contain these two domains in the same module in the query builder.

The antiSMASH database has been expanded to cover more than just bacterial genomes. It now contains 147 517 high-quality BGCs from 388 archaeal, 25 236 bacterial, and 177 fungal representative high-quality genomes. Annotations were generated by antiSMASH 5.2, the most recent version. antiSMASH 5 added detection rules for *N*-acyl amino acids, β-lactones, polybrominated diphenyl ethers, C-nucleosides, pseudopyronines, fungal RiPPs, RaS-RiPPs, TfuA-related RiPPs, and lanthidines. antiSMASH 5 also can predict type II PKS cluster products in more detail, gives better information on BGC regions potentially containing multiple clusters in close vicinity, and a cleaned up user interface. Version 3 of the database of course makes all of these new BGC types available (see Figure 1A, B). On top of these new features described in more detail in the antiSMASH 5 publication (13), antiSMASH gained a major new analysis in version 5.1: it now predicts the biosynthetic modules that make up modular NRPS and modular type I PKS clusters. Instead of just predicting the substrates activated by the respective loading modules, detected modifications such as epimerization, reduction and dehydration can now be applied to the loaded substrate to predict the final monomer added to the produced compound. This new antiSMASH feature is mirrored by a new query type in the antiSMASH database. The module query builder allows querying the database for clusters containing modules with user-specified domains, allowing searches like ‘Find clusters containing a *trans*-acyltransferase PKS module with a dehydratase and a carbon methyltransferase’ (see Figure 1C). All query types now save the query in the browser's URL bar, making it possible to save queries or to share queries with collaborators.

While this version of the database only sees a slight increase of covered bacterial genomes (∼2%), it is the first version to also cover Archaea and Fungi. Additionally, the quality of the genome assemblies has improved. Version 3 contains 169 181 regions with BGCs compared to version 2’s 152 106 (up ∼11%), while decreasing the number of BGCs starting or ending at a contig edge (21 664 in v3, compared to 41 882 in v2, down ∼48%). When BGCs are in contact with a contig edge, they are likely fragmented across multiple contigs; this is not the case for 147 517 regions. In Archaea, 820 out of 853 BGC regions (∼96%) are not fragmented. In Bacteria 143 561 out of 165 084 (∼87%) of the BGC regions are not fragmented. In Fungi, 3136 out of 3244 (∼97%) of BGC regions are not located at a contig edge. The difference in percentages is probably caused by the higher percentage of Bacteria carrying highly repetitive multimodular BGCs, such as modular nonribosomal peptide synthases (NRPS) and type I modular PKS, that are more likely to cause assembly errors on short read sequencing data (22). Indeed, only 31 regions in Archaea contain modular NRPS BGCs, and none contain PKS type I BGCs. In Fungi, while more NRPS and PKS type I BGC regions are present (817 NRPS and 1065 PKS type I), the clusters tend to be smaller and thus less repetitive and less likely to be affected by contig breaks. The largest fungal BGC region containing a modular NRPS also contains a PKS type I BGC and is ∼130 kbp in size. In contrast the largest bacterial BGC region, also containing both a modular NRPS and a PKS type I BGC, is ∼391 kb. Even on average, bacterial NRPS regions are larger than the fungal ones (∼57 kb in bacteria, ∼55 kb in fungi). The difference is even more pronounced in PKS type I clusters (∼61 kb in bacteria, ∼51 kb in fungi). These differences exist even though bacterial genomes tend to pack genes much more tightly, whereas fungal genomes have larger intergenic distances.

## CONCLUSIONS

Genome mining continues to be a valuable methodology for assessing microbial biosynthetic potential. These efforts have been aided by antiSMASH since 2011. With >750 000 jobs processed on the public web server, and >25 000 downloads of the standalone version, antiSMASH is one of the tools of choice in the natural product field. The antiSMASH database helps to compare identified clusters across genomes and allows for more complex searches to contextualise and cross-reference findings via a user-friendly web interface.

With a selection of 147 517 BGC regions from Archaea, Bacteria and Fungi, version 3 of the antiSMASH database is a comprehensive and highly integrated collection of secondary/specialized metabolite biosynthetic gene clusters with up-to-date, high quality antiSMASH-based annotations available to the natural product research community.

## DATA AVAILABILITY

The antiSMASH database is available at https://antismash-db.secondarymetabolites.org/. There are no access restrictions for academic or commercial use of the web server. The source code components and SQL schema for the antiSMASH database are available on GitHub (https://github.com/antismash) under an OSI-approved Open Source license.
